# Association Between Post-Traumatic Stress and Depression Among Older Puerto Ricans: How Does Loneliness Matter?

**DOI:** 10.1093/geroni/igab046.1583

**Published:** 2021-12-17

**Authors:** Kaipeng Wang, Adolfo Cuevas, Anao Zhang, Carson De Fries, Ladson Hinton, Luis Falcón

**Affiliations:** 1 University of Denver, Denver, Colorado, United States; 2 Tufts University, Medford, Massachusetts, United States; 3 University of Michigan, Ann Arbor, Michigan, United States; 4 University of California, Davis, Sacramento, California, United States; 5 University of Massachusetts Lowell, Lowell, Massachusetts, United States

## Abstract

Puerto Ricans have a significantly higher prevalence of post-traumatic stress disorder (PTSD) than other racial and ethnic groups. PTSD can lead to co-morbid depression, which exacerbates the risk for compromised mental and physical health among older adults. Loneliness, a robust indicator of poor social support, is likely to worsen the dysphoric symptoms of PTSD and increase the risks of depression. However, few studies have focused on the moderating role of loneliness on the association between post-traumatic stress and depression. This study examines the association between post-traumatic stress and depression and whether such an association differs by loneliness among older Puerto Ricans. Data were collected from 304 Puerto Ricans aged 60-81 living in the Greater Boston area who responded to questionnaires in a survey. We used ordinary least squares regression to examine the association between post-traumatic stress, loneliness, and depression. We found that post-traumatic stress was significantly associated with higher levels of depression; however, the association was contingent on experiences of loneliness. The association between post-traumatic stress and depression was stronger for those experiencing a higher degree of loneliness. Findings underscored the role that loneliness plays in exacerbating the risk of developing co-morbid PTSD and depression among older Puerto Ricans. In working with older Puerto Ricans experiencing post-traumatic stress, it is important for mental health professionals to incorporate the assessment of loneliness and to prevent and reduce co-morbid depression by addressing loneliness through improving social skills, enhancing social support, and reducing maladaptive social cognition.

